# Supplementary material for: A direct approach to estimating false discovery rates conditional on covariates
Source: PeerJ. 2018 Dec 10;6:e6035. doi: 10.7717/peerj.6035 (PMC6292380; doi:10.7717/peerj.6035)

Supplementary Materials for "A direct approach to estimating false discovery rates conditional on covariates"

Simina M. Boca and Jeffrey T. Leek

October 15, 2018

S1 Derivation of Algorithm 1

We assume that a hypothesis test is performed for each i, summarized by a p-value P_i. Our approach is based on thresholding the p-values at a given $\lambda \in (0, 1)$, resulting in binary indicators $Y_i = 1(P_i > \lambda)$. These are then treated as outcomes in a regression model.

Since Y_i is a dichotomous random variable that is 1 when the null hypothesis H_{0i} is not rejected at a significance level of λ and 0 when it is rejected, $m - R = \sum_{i=1}^{m} Y_i$ for a fixed, given λ. The null p-values will come from a Uniform(0,1) distribution, while the p-values for the features from the alternative distributions $G_{\mathbf{x}_i}$, defined as:

$$G_{\mathbf{x}_i}(\lambda) = Pr(P_i \leq \lambda | \theta_i = 1, \mathbf{X}_i = \mathbf{x}_i). \quad (1)$$

The major assumption we make moving forward is that *conditional on the null, the p-values do not depend on the covariates.*

Theorem S1 *Suppose that m hypotheses tests are performed and that conditional on the null, the p-values do not depend on the covariates. Furthermore, the null p-values have a Uniform(0,1) distribution, whereas the alternative p-values have a distribution with cdf $G_{\mathbf{x}_i}$, as defined above. Then:*

$$E(Y_i|\mathbf{X}_i = \mathbf{x}_i) \quad = \quad (1 - \lambda)\pi_0(\mathbf{x}_i) + \{1 - G_{\mathbf{x}_i}(\lambda)\}\{1 - \pi_0(\mathbf{x}_i)\}.$$

We first review the algorithm which yields an estimator of π_0 for the no-covariate case, which is used by Storey (2002), then develop a procedure based on Theorem S1 to obtain an estimator of $\pi_0(\mathbf{x}_i)$. Both of them are based on

assuming reasonably powered tests and a large enough λ, so that

$$G_{\mathbf{x}_i}(\lambda) \approx 1.$$

Theorem S1 can then be applied assuming no covariates, leading to:

$$\pi_0 \approx \frac{E(Y_i)}{1-\lambda},$$

resulting in:

$$\pi_0 \approx \frac{\frac{\sum_{i=1}^m E(Y_i)}{m}}{1-\lambda}.$$

Using a method-of-moments approach, one may consider the estimator:

$$\hat{\pi}_0 = \frac{\frac{\sum_{i=1}^m Y_i}{m}}{1-\lambda} = \frac{m-R}{(1-\lambda)m}, \quad (2)$$

which is used by Storey (2002).

For the GWAS meta-analysis dataset, using this approach with $\lambda = 0.8$ leads to an $\hat{\pi}_0 = 0.951$ and $\lambda = 0.9$ to $\hat{\pi}_0 = 0.949$. Note that in practice one may smooth over a series of thresholds, as described below; otherwise, fixed thresholds between 0.8 and 0.95 are often used. This means that $G_{\mathbf{x}_i}(\lambda)$ will be very close to 1, but λ will not be large enough to lead to numerical instability issues when dividing by $1-\lambda$.

For the covariate case, applying the same steps with Theorem S1, we get:

$$\pi_0(\mathbf{x}_i) \approx \frac{E(Y_i|\mathbf{X}_i = \mathbf{x}_i)}{1-\lambda}.$$

We can use a regression framework to estimate $E(Y_i|\mathbf{X}_i = \mathbf{x}_i)$, then estimate $\pi_0(\mathbf{x})$ by:

$$\hat{\pi}_0(\mathbf{x}_i) = \frac{\hat{E}(Y_i|\mathbf{X}_i = \mathbf{x}_i)}{1-\lambda}, \quad (3)$$

obtaining Step (c) in the algorithm.

Note that thus far we have considered the estimate of $\pi_0(\mathbf{x}_i)$ at a single threshold λ, so that $\hat{\pi}_0(\mathbf{x}_i)$ is in fact $\hat{\pi}_0^{\lambda}(\mathbf{x}_i)$. We generally prefer to smooth over a series of thresholds to obtain the final estimate, as done by Storey and Tibshirani

(2003). The estimates should generally be thresholded at 1, as Eq. (3) may otherwise lead to values greater than 1. It is also possible but less likely that the smoothed estimate would be below 0, hence we also threshold it at 0. If we assume that the p-values are independent, we can also use bootstrap samples of them to obtain a confidence interval for $\hat{\pi}_0(\mathbf{x}_i)$ - Steps (e) and (f) in Algorithm 1.

In order to obtain Step (g) in the algorithm and estimate $\text{FDR}(\mathbf{x}_i)$, we multiply the BH adjusted p-values by $\hat{\pi}_0(\mathbf{x}_i)$, thus leading to a simple plug-in estimator, denoted $\widehat{\text{FDR}}(\mathbf{x}_i)$. This is done in the spirit of Storey (2002), whose approach uses an estimate which is not conditional on covariates.

S2 Special cases

S2.1 No covariates

If we do not consider any covariates, the usual estimator $\hat{\pi}_0$ from Eq. (2) can be deduced from applying Algorithm 1 by fitting a linear regression with just an intercept.

S2.2 Partioning the features

Now assume that the set of m features is partitioned into S sets, namely that a collection of sets $\mathcal{S} = \{A_s : 1 \leq s \leq S\}$ is considered such that all sets are non-empty, pairwise disjoint, and have the set of all the features as their union. This idea has been proposed before, for example in Hu et al. (2010), but we propose it here as a natural subcase of our approach. We consider the sets ordered for the sake of convenience, for example, the first set in $\mathcal{S}$ is A_1 et cetera, but note that this ordering does not necessarily have scientific relevance. In the GWAS meta-analysis dataset, the SNPs are partitioned according to their MAFs. Other examples of such partionings include all possible atoms resulting from gene-set annotations or brain regions of interest in a functional imaging analysis, when considering only the genes or voxels that are annotated (Boca et al., 2013). We then consider vectors $\mathbf{x}_i$ of length S, $1 \leq i \leq m$, such that element s of $\mathbf{x}_i$ is defined, using the indicator notation, as:

$$x_{is} = \begin{cases} 1 \text{ if } i \in A_s, \\ 0 \text{ if } i \notin A_s. \end{cases} \tag{4}$$

For example, if $S = 3$ and feature 1 was in set A_1, then $\mathbf{x}_1 = (1, 0, 0)'$. Since all features i in a set A_s have the same vector $\mathbf{x}_i$, we denote it by $\mathbf{e}_{A_s}$ to emphasize this. Taking into account the partition, a natural way of estimating $\pi_0(\mathbf{e}_{A_s})$

is to just apply the estimator $\hat{\pi}_0$ from Eq. (2) to each of the S sets:

$$\hat{\pi}_0(\mathbf{e}_{A_s}) = \frac{\frac{\sum_{i \in A_s} Y_i}{|A_s|}}{1-\lambda} \text{ for } 1 \leq s \leq S-1,$$

where the numerator $\frac{\sum_{i \in A_s} Y_i}{|A_s|}$ represents the fraction of features in A_s that are not discoveries at the λ threshold.

A related idea has been proposed for partitioning hypotheses into sets to improve power (Efron, 2008). These results would be obtained directly from our approach if we considered linear instead of logistic regression and fit a linear regression with no intercept and the covariates $\mathbf{x}_i$ in Algorithm 1, or instead, set one of the sets as the baseline and also considered an intercept. As we are considering a logistic regression approach, our results will be slightly different.

S3 Theoretical results

We now proceed to explore some theoretical properties of the estimator $\hat{\pi}_0^\lambda(\mathbf{x}_i)$. Applying Theorem S1 to Eq. (3), we get that:

$$\hat{\pi}_0^\lambda(\mathbf{x}_i) = \pi_0(\mathbf{x}_i) + \frac{1 - G_{\mathbf{x}_i}(\lambda)}{1-\lambda}\{1 - \pi_0(\mathbf{x}_i)\} + \frac{b(\mathbf{x}_i)}{1-\lambda}, \tag{5}$$

where $b(\mathbf{x}_i) = \hat{E}(Y_i|\mathbf{X}_i = \mathbf{x}_i) - E(Y_i|\mathbf{X}_i = \mathbf{x}_i)$, so that $E\{b(\mathbf{x}_i)\}$ is the bias of $\hat{E}(Y_i|\mathbf{X}_i = \mathbf{x}_i)$ when estimating $E(Y_i|\mathbf{X}_i = \mathbf{x}_i)$. Note that $\frac{1-G_{\mathbf{x}_i}(\lambda)}{1-\lambda}\{1 - \pi_0(\mathbf{x}_i)\} \geq 0$, since $\lambda \leq 1, G_{\mathbf{x}_i}(\lambda) \leq 1$, and $\pi_0(\mathbf{x}_i) \leq 1$. Thus, if the bias when estimating $E(Y_i|\mathbf{X}_i = \mathbf{x}_i)$ is positive or negative and small in absolute value, then $\hat{\pi}_0^\lambda(\mathbf{x}_i)$ is a conservative estimator of $\pi_0(\mathbf{x}_i)$. For example, if we had considered a correctly specified linear regression model, this would always hold; indeed the case where π_0 is shared by all the features, i.e. in the case of no dependence on covariates, this is shown in Storey (2002). Given that here we are taking $\hat{E}(Y_i|\mathbf{X}_i = \mathbf{x}_i)$ to be the MLE from the logistic regression model, we know that it represents a consistent estimator of $E(Y_i|\mathbf{X}_i = \mathbf{x}_i)$ if the model is correctly specified for $m \to \infty$, given certain technical conditions, for instance those specified in Gourieroux and Monfort (1981). Thus, we can show that $\hat{\pi}_0^\lambda(\mathbf{x}_i)$ is a consistent estimator of $\pi_0(\mathbf{x}_i) + \frac{1-G_{\mathbf{x}_i}(\lambda)}{1-\lambda}\{1 - \pi_0(\mathbf{x}_i)\}$ under these same conditions:

Theorem S2 *Under a correctly specified model and technical regularity conditions,*

$$\hat{\pi}_0^\lambda(\mathbf{x}_i) \to_p \pi_0(\mathbf{x}_i) + \frac{1 - G_{\mathbf{x}_i}(\lambda)}{1-\lambda}\{1 - \pi_0(\mathbf{x}_i)\} \geq \pi_0(\mathbf{x}_i).$$

as $m \to \infty$.

Eq. (5) also leads to Var$\{\hat{\pi}_0^\lambda(\mathbf{x}_i)\} = \frac{Var\{b(\mathbf{x}_i)\}}{(1-\lambda)^2}$. Once again, using the properties of the MLE, under appropriate conditions:

$$\sqrt{m} b(\mathbf{x}_i) \to_D N(0, \sigma^2)$$

for some σ^2, leading to $Var\{\hat{\pi}_0^\lambda(\mathbf{x}_i)\}$ being approximately inversely proportional to m for large values of m.

We note that our approach to estimating $\pi_0(\mathbf{x}_i)$ does not place any restrictions on its range. In practice, the values will also be thresholded to be between 0 and 1, as detailed in Algorithm 1. In Result S3, we show that implementing this thresholding decreases the mean squared error of the estimator. The approach is similar to that taken in Theorem 2 in the work of Storey (2002).

Result S3 *Let*

$$\hat{\pi}_0^C(\mathbf{x}_i) = \begin{cases} 0 & \hat{\pi}_0(\mathbf{x}_i) < 0 \\ \hat{\pi}_0(\mathbf{x}_i) & 0 \leq \hat{\pi}_0(\mathbf{x}_i) \leq 1 \\ 1 & 1 < \hat{\pi}_0(\mathbf{x}_i) \end{cases}$$

Then:

$$E[\{\hat{\pi}_0(\mathbf{x}_i) - \pi_0(\mathbf{x}_i)\}^2] \geq E[\{\hat{\pi}_0^C(\mathbf{x}_i) - \pi_0(\mathbf{x}_i)\}^2].$$

S4 Proofs of analytical results

Proof of Theorem S1

$$\begin{aligned} E(Y_i|\mathbf{X}_i = \mathbf{x}_i) &= Pr(P_i > \lambda|\mathbf{X}_i = \mathbf{x}_i) \\ &= Pr(P_i > \lambda|\theta_i = 0, \mathbf{X}_i = \mathbf{x}_i)P(\theta_i = 0|\mathbf{X}_i = \mathbf{x}_i) \\ &+ Pr(P_i > \lambda|\theta_i = 1, \mathbf{X}_i = \mathbf{x}_i)P(\theta_i = 1|\mathbf{X}_i = \mathbf{x}_i). \end{aligned}$$

Then, using the assumption that conditional on the null, the p-values do not depend on the covariates:

$$
\begin{aligned}
E(Y_i|\mathbf{X}_i = \mathbf{x}_i) &= Pr(P_i > \lambda|\theta_i = 0)P(\theta_i = 0|\mathbf{X}_i = \mathbf{x}_i) \\
&+ Pr(P_i > \lambda|\theta_i = 1, \mathbf{X}_i = \mathbf{x}_i)P(\theta_i = 1|\mathbf{X}_i = \mathbf{x}_i) \\
&= (1-\lambda)\pi_0(\mathbf{x}_i) + \{1 - G_{\mathbf{x}_i}(\lambda)\}\{1 - \pi_0(\mathbf{x}_i)\}.
\end{aligned}
$$

Proof of Result S3

We prove this result by showing that:

$$
E[\{\hat{\pi}_0(\mathbf{x}_i) - \pi_0(\mathbf{x}_i)\}^2|\hat{\pi}_0(\mathbf{x}_i) > 1] > E[\{\hat{\pi}_0(\mathbf{x}_i)^C - \pi_0(\mathbf{x}_i)\}^2|\hat{\pi}_0(\mathbf{x}_i) > 1] \tag{6}
$$

and:

$$
E[\{\hat{\pi}_0(\mathbf{x}_i) - \pi_0(\mathbf{x}_i)\}^2|\hat{\pi}_0(\mathbf{x}_i) < 0] > E[\{\hat{\pi}_0^C(\mathbf{x}_i) - \pi_0(\mathbf{x}_i)\}^2|\hat{\pi}_0(\mathbf{x}_i) < 0]. \tag{7}
$$

Then, we can combine them as follows:

$$
\begin{aligned}
& E[\{\hat{\pi}_0(\mathbf{x}_i) - \pi_0(\mathbf{x}_i)\}^2] - E[\{\hat{\pi}_0^C(\mathbf{x}_i) - \pi_0(\mathbf{x}_i)\}^2] = \\
= \; & E[\{\hat{\pi}_0(\mathbf{x}_i) - \pi_0(\mathbf{x}_i)\}^2|\hat{\pi}_0(\mathbf{x}_i) > 1] - E[\{\hat{\pi}_0(\mathbf{x}_i)^C - \pi_0(\mathbf{x}_i)\}^2|\hat{\pi}_0(\mathbf{x}_i) > 1]P\{\hat{\pi}_0(\mathbf{x}_i) > 1\} \\
+ \; & E[\{\hat{\pi}_0(\mathbf{x}_i) - \pi_0(\mathbf{x}_i)\}^2|\hat{\pi}_0(\mathbf{x}_i) < 0] - E[\{\hat{\pi}_0^C(\mathbf{x}_i) - \pi_0(\mathbf{x}_i)\}^2|\hat{\pi}_0(\mathbf{x}_i) < 0]P\{\hat{\pi}_0(\mathbf{x}_i) < 0\} \\
\geq \; & 0.
\end{aligned}
$$

In Eq. (6):

$$
\begin{aligned}
& E[\{\hat{\pi}_0(\mathbf{x}_i) - \pi_0(\mathbf{x}_i)\}^2|\hat{\pi}_0(\mathbf{x}_i) > 1] - E[\{\hat{\pi}_0^C(\mathbf{x}_i) - \pi_0(\mathbf{x}_i)\}^2|\hat{\pi}_0(\mathbf{x}_i) > 1] = \\
= \; & E[\{\hat{\pi}_0(\mathbf{x}_i) - 1\}\{\hat{\pi}_0(\mathbf{x}_i) + 1 - 2\pi_0(\mathbf{x}_i)\}|\hat{\pi}_0(\mathbf{x}_i) > 1] > 0,
\end{aligned}
$$

because in this region $\hat{\pi}_0(\mathbf{x}_i) + 1 > 2 \geq 2\pi_0(\mathbf{x}_i)$.

In Eq. (7):

$$E[\{\hat{\pi}_0(\mathbf{x}_i) - \pi_0(\mathbf{x}_i)\}^2|\hat{\pi}_0(\mathbf{x}_i) < 0] - E[\{\hat{\pi}_0^C(\mathbf{x}_i) - \pi_0(\mathbf{x}_i)\}^2|\hat{\pi}_0(\mathbf{x}_i) < 0] =$$

$$= E[\{a - \hat{\pi}_0(\mathbf{x}_i)\}\{2\pi_0(\mathbf{x}_i) - \hat{\pi}_0(\mathbf{x}_i) - 0\}|\hat{\pi}_0(\mathbf{x}_i) < 0] > 0,$$

because in this region $2\pi_0(\mathbf{x}_i) \geq 0 > \hat{\pi}_0(\mathbf{x}_i)$.

S5 Functions $\pi_0(\mathbf{x}_i)$ used in simulation scenarios

Below, we refer to scenarios I-IV, as in Figure 3:

In scenarios I-IV, the values of x_1 are equally spaced between 0 and 1, with the number of points being equal to m, the number of features considered.

- Scenario I: $\pi_0(x_1) = 0.9$
- Scenario II: $\pi_0(x_1) = \pi_{01}(x_1) + \pi_{02}(x_1) + 0.12\pi_{03}(x_1)$, where:

$$\pi_{01}(x_1) = \begin{cases} 1 \text{ if } 0 \leq x_1 \leq 0.5 \\ -4/1.96(x_1 + 0.2)(x_1 - 1.2) \text{ if } 0.5 < x_1 < 0.7 \\ 4/1.96 \times 0.45 \text{ if } 0.7 \leq x_1 \leq 1, \end{cases}$$

$$\pi_{02}(x_1) = \begin{cases} 0 \text{ if } 0 \leq x_1 < 0.7 \\ -2.5(x - 0.7)^2 \text{ if } 0.7 \leq x_1 \leq 1 \end{cases}$$

$$\pi_{03}(x_1) = \begin{cases} 0 \text{ if } 0 \leq x_1 \leq 0.1 \\ -(x - 0.1)^2 \text{ if } 0.1 < x_1 < 0.7 \\ -0.36 \text{ if } 0.7 \leq x_1 \leq 1. \end{cases}$$

- Scenario III:

$$\pi_0(x_1, x_2) = \begin{cases} \pi_{01}(x_1) + \pi_{02}(x_1) + 0.12\pi_{03}(x_1) \text{ if } x_2 = 1 \\ \pi_{01}(x_1) + 0.5\pi_{02}(x_1) + 0.06\pi_{03}(x_1) \text{ if } x_2 = 2 \\ \pi_{01}(x_1) + 0.3\pi_{02}(x_1) \text{ if } x_2 = 3, \end{cases}$$

where x_2 is defined by first randomly generating m points from Unif$(0, 0.5)$, then creating discrete categories by using the thresholds 0.127 and 0.302 and $\pi_{01}, \pi_{02}, \pi_{03}$ are defined as in Scenario II.

- Scenario IV: $\pi_0(x_1, x_2)$ is the same function as in scenario III multiplied by 0.6.

- Scenario V: $\pi_0(x_1) = x_1$

References

Boca, S. M., Corrada Bravo, H., Caffo, B., Leek, J. T., and Parmigiani, G. (2013). A decision-theory approach to interpretable set analysis for high-dimensional data. *Biometrics* doi: 10.1111/biom.12060.

Efron, B. (2008). Simultaneous inference: When should hypothesis testing problems be combined? *The Annals of Applied Statistics* pages 197–223.

Gourieroux, C. and Monfort, A. (1981). Asymptotic properties of the maximum likelihood estimator in dichotomous logit models. *Journal of Econometrics* **17,** 83–97.

Hu, J. X., Zhao, H., and Zhou, H. H. (2010). False discovery rate control with groups. *Journal of the American Statistical Association* **105,** 1215–1227.

Storey, J. D. (2002). A direct approach to false discovery rates. *Journal of the Royal Statistical Society: Series B (Statistical Methodology)* **64,** 479–498.

Storey, J. D. and Tibshirani, R. (2003). Statistical significance for genomewide studies. *Proceedings of the National Academy of Sciences* **100,** 9440–9445.

S6 Supplementary tables

Table S1: Results for BMI GWAS meta-analysis giving the number of SNPs with an estimated FDR $\leq 5\%$ for various approaches. BL = Boca-Leek, Scott T = Scott theoretical null, Scott E = Scott empirical null, BH = Benjamini-Hochberg.

	BL	Scott T	Scott E	Storey	BH
Number with $\widehat{FDR} \leq 5\%$	13384	16697	7636	12771	12500

Table S2: Simulation results for $m = 1,000$ features, 200 runs for each scenario, independent test statistics. "Reg. model" = specific logistic regression model considered, BL = Boca-Leek, Scott T = Scott theoretical null, Scott E = Scott empirical null, BH = Benjamini-Hochberg. A nominal FDR = 5% was considered. Results for the Scott approaches are only presented for scenarios which generate z-statistics or t-statistics.

			FDR %					TPR %				
$\pi_0(x)$	Dist. under H_1	Reg. model	BL	Scott T	Scott E	Storey	BH	BL	Scott T	Scott E	Storey	BH
I	Beta(1,20)	Spline	5.0			5.2	3.9	0.2			0.2	0.1
V	Beta(1,20)	Spline	3.5			4.9	3.1	66.6			20.6	0.4
I	Norm	Spline	5.1	5.5	6.7	4.9	4.4	51.2	51.1	50.0	50.8	49.7
V	Norm	Spline	4.7	4.9	24.9	4.7	2.4	80.5	83.4	74.1	74.1	67.1
I	T	Spline	6.0	22.8	24.3	5.5	4.8	16.1	48.7	50.0	15.2	13.6
V	T	Spline	4.5	7.6	9.4	4.7	2.5	68.3	80.5	50.7	57.1	43.3
I	Chisq 1 df	Spline	5.0			4.8	4.4	51.2			50.9	49.7
V	Chisq 1 df	Spline	4.4			4.8	2.5	78.9			73.9	66.8
I	Chisq 4 df	Spline	5.3			5.4	4.8	30.8			30.6	29.6
V	Chisq 4 df	Spline	4.0			4.6	2.4	62.8			55.3	46.2

Table S3: Simulation results for $m = 1,000$ features, 200 runs per scenario, dependent test statistics from a multivariate normal distribution with a block-diagonal variance-covariance matrix. B = block size, ρ = within-block correlation. "Reg. model" = specific logistic regression model considered, BL = Boca-Leek, Scott T = Scott theoretical null, Scott E = Scott empirical null, BH = Benjamini-Hochberg. Nominal FDR = 5%.

			FDR %					TPR %				
$\pi_0(x)$	Dist. under H_1	Reg. model	BL	Scott T	Scott E	Storey	BH	BL	Scott T	Scott E	Storey	BH
I	N, B=20, ρ=0.2	Linear	5.3	6.2	6.8	5.0	4.4	51.5	51.4	48.4	51.3	50.1
II	N, B=20, ρ=0.2	Linear	5.2	6.9	8.0	5.1	4.6	48.6	63.4	59.3	47.6	46.5
II	N, B=20, ρ=0.2	Spline	5.7	8.3	9.2	5.1	4.6	49.2	63.3	59.6	47.6	46.5
III	N, B=20, ρ=0.2	Linear	5.5	7.6	9.3	5.2	4.8	45.1	60.0	56.0	44.0	43.2
III	N, B=20, ρ=0.2	Spline	5.7	9.6	10.6	5.2	4.8	45.9	60.2	56.3	44.0	43.2
IV	N, B=20, ρ=0.2	Linear	5.3	5.3	2.5	4.9	2.9	71.8	71.9	61.0	71.4	65.6
IV	N, B=20, ρ=0.2	Spline	5.6	5.5	2.5	4.9	2.9	72.0	71.9	61.1	71.4	65.6
V	N, B=20, ρ=0.2	Linear	4.5	5.2	23.2	4.8	2.5	79.0	83.0	74.6	74.1	66.9
I	N, B=20, ρ=0.5	Linear	6.4	10.0	10.7	6.0	5.2	52.0	51.7	47.6	51.6	50.3
II	N, B=20, ρ=0.5	Linear	6.1	12.4	13.5	5.7	5.1	48.4	62.8	57.6	47.3	46.2
II	N, B=20, ρ=0.5	Spline	7.1	18.7	20.4	5.7	5.1	49.5	62.6	58.0	47.3	46.2
III	N, B=20, ρ=0.5	Linear	5.6	11.5	15.9	5.2	4.6	45.4	59.6	56.6	44.0	43.2
III	N, B=20, ρ=0.5	Spline	6.6	19.9	23.6	5.2	4.6	46.2	59.0	56.9	44.0	43.2
IV	N, B=20, ρ=0.5	Linear	5.8	6.1	2.8	5.3	3.1	72.1	72.3	59.4	71.6	65.7
IV	N, B=20, ρ=0.5	Spline	6.5	6.4	3.0	5.3	3.1	72.4	72.2	59.6	71.6	65.7
V	N, B=20, ρ=0.5	Linear	4.6	6.0	22.4	4.9	2.4	79.2	83.2	72.1	74.3	66.9
I	N, B=20, ρ=0.9	Linear	9.0	17.6	36.2	6.9	5.3	53.8	53.3	57.9	52.6	50.4
II	N, B=20, ρ=0.9	Linear	7.8	20.0	47.5	6.4	4.9	49.6	63.8	68.0	48.0	46.2
II	N, B=20, ρ=0.9	Spline	18.2	34.5	53.6	6.4	4.9	52.2	64.4	69.8	48.0	46.2
III	N, B=20, ρ=0.9	Linear	6.4	23.1	48.8	5.1	4.0	47.3	60.5	67.9	46.1	44.0
III	N, B=20, ρ=0.9	Spline	21.5	38.4	60.5	5.1	4.0	51.0	60.9	69.7	46.1	44.0
IV	N, B=20, ρ=0.9	Linear	7.7	8.4	6.9	6.1	3.1	73.1	73.2	57.4	72.2	65.9
IV	N, B=20, ρ=0.9	Spline	11.8	10.0	8.0	6.1	3.1	74.4	72.8	57.8	72.2	65.9
V	N, B=20, ρ=0.9	Linear	5.5	7.9	22.1	5.3	2.2	79.1	83.7	69.9	74.5	66.8
I	N, B=10, ρ=0.2	Linear	5.4	7.8	6.1	5.1	4.4	51.6	51.6	47.3	51.2	49.9
II	N, B=10, ρ=0.2	Linear	5.0	9.3	8.8	4.8	4.3	48.2	63.0	59.8	47.2	46.1
II	N, B=10, ρ=0.2	Spline	5.5	13.3	11.1	4.8	4.3	49.1	62.8	59.8	47.2	46.1
III	N, B=10, ρ=0.2	Linear	5.2	8.6	9.8	5.0	4.5	44.6	59.5	56.4	43.4	42.7
III	N, B=10, ρ=0.2	Spline	5.8	14.3	13.2	5.0	4.5	45.2	59.2	56.6	43.4	42.7
IV	N, B=10, ρ=0.2	Linear	5.3	5.7	2.4	5.0	2.9	71.8	71.8	60.4	71.4	65.5
IV	N, B=10, ρ=0.2	Spline	5.7	5.9	2.5	5.0	2.9	72.1	71.8	60.5	71.4	65.5
V	N, B=10, ρ=0.2	Linear	4.4	5.3	21.6	4.8	2.5	78.5	82.8	73.0	73.8	66.7
I	N, B=10, ρ=0.5	Linear	7.3	17.1	15.9	6.5	5.4	51.9	51.8	48.8	51.7	50.0
II	N, B=10, ρ=0.5	Linear	5.9	20.3	19.9	5.3	4.5	48.3	62.6	61.0	46.8	45.6
II	N, B=10, ρ=0.5	Spline	8.6	32.5	27.7	5.3	4.5	49.2	63.3	61.4	46.8	45.6
III	N, B=10, ρ=0.5	Linear	5.8	17.4	17.7	4.9	4.2	44.2	58.1	54.3	43.0	42.0
III	N, B=10, ρ=0.5	Spline	8.6	32.7	30.2	4.9	4.2	45.0	58.1	55.6	43.0	42.0
IV	N, B=10, ρ=0.5	Linear	6.3	7.5	3.3	5.5	3.2	72.4	72.4	59.0	71.9	65.8
IV	N, B=10, ρ=0.5	Spline	7.6	8.3	3.8	5.5	3.2	72.7	72.1	59.3	71.9	65.8
V	N, B=10, ρ=0.5	Linear	4.7	6.5	20.4	4.9	2.3	78.6	83.2	69.2	73.8	66.5
I	N, B=10, ρ=0.9	Linear	14.1	30.6	45.6	6.6	4.1	55.5	54.7	65.6	53.3	50.2
II	N, B=10, ρ=0.9	Linear	13.3	35.5	55.9	5.9	3.3	51.1	66.5	75.8	49.0	46.1
II	N, B=10, ρ=0.9	Spline	35.1	49.9	67.5	5.9	3.3	56.1	67.4	77.6	49.0	46.1
III	N, B=10, ρ=0.9	Linear	13.3	33.7	66.4	5.4	3.3	45.6	58.1	75.7	43.4	40.7
III	N, B=10, ρ=0.9	Spline	40.7	51.5	73.0	5.4	3.3	52.0	61.6	77.4	43.4	40.7
IV	N, B=10, ρ=0.9	Linear	11.2	12.4	12.0	7.0	3.1	74.0	73.5	63.9	72.5	65.8
IV	N, B=10, ρ=0.9	Spline	19.2	15.6	13.8	7.0	3.1	76.2	73.3	64.3	72.5	65.8
V	N, B=10, ρ=0.9	Linear	7.1	10.3	21.9	6.0	2.1	79.6	84.2	67.5	74.7	66.3

Table S4: Simulation results for $m = 1,000$ features, 200 runs per scenario, dependent test statistics from a multivariate t distribution with a block-diagonal variance-covariance matrix. B = block size, ρ = within-block correlation. "Reg. model" = specific logistic regression model considered, BL = Boca-Leek, Scott T = Scott theoretical null, Scott E = Scott empirical null, BH = Benjamini-Hochberg. Nominal FDR = 5%.

			FDR %					TPR %				
$\pi_0(x)$	Dist. under H_1	Reg. model	BL	Scott T	Scott E	Storey	BH	BL	Scott T	Scott E	Storey	BH
I	T, B=20, ρ=0.2	Linear	1.7	9.1	7.4	1.5	0.9	8.0	51.6	57.8	7.6	5.7
II	T, B=20, ρ=0.2	Linear	3.2	13.9	7.3	3.2	1.8	8.0	63.8	61.0	6.8	4.5
II	T, B=20, ρ=0.2	Spline	3.7	14.7	8.5	3.2	1.8	9.2	63.9	61.3	6.8	4.5
III	T, B=20, ρ=0.2	Linear	2.6	13.8	9.6	2.1	1.3	4.3	59.4	60.1	3.4	2.3
III	T, B=20, ρ=0.2	Spline	3.6	15.1	11.0	2.1	1.3	5.2	59.7	60.3	3.4	2.3
IV	T, B=20, ρ=0.2	Linear	2.7	5.4	2.9	2.4	1.0	55.4	71.8	65.1	54.4	44.3
IV	T, B=20, ρ=0.2	Spline	3.0	5.4	2.8	2.4	1.0	56.0	71.9	65.1	54.4	44.3
V	T, B=20, ρ=0.2	Linear	2.9	5.6	23.9	3.1	1.2	70.3	82.8	71.3	60.8	48.1
I	T, B=20, ρ=0.5	Linear	1.7	10.3	11.0	1.5	1.0	8.6	51.6	57.4	8.2	5.9
II	T, B=20, ρ=0.5	Linear	3.5	16.3	11.9	3.3	2.1	7.7	64.2	61.7	6.6	4.5
II	T, B=20, ρ=0.5	Spline	4.7	19.5	16.6	3.3	2.1	9.1	63.9	62.1	6.6	4.5
III	T, B=20, ρ=0.5	Linear	3.2	17.6	13.0	2.3	1.5	5.0	59.3	59.0	3.6	2.6
III	T, B=20, ρ=0.5	Spline	4.4	23.4	20.5	2.3	1.5	5.6	59.6	59.5	3.6	2.6
IV	T, B=20, ρ=0.5	Linear	2.7	5.5	3.0	2.3	1.0	55.3	71.9	64.7	54.3	44.4
IV	T, B=20, ρ=0.5	Spline	3.2	5.8	3.1	2.3	1.0	55.8	71.9	64.8	54.3	44.4
V	T, B=20, ρ=0.5	Linear	3.1	6.2	23.0	3.1	1.2	69.4	82.6	69.4	59.9	47.2
I	T, B=20, ρ=0.9	Linear	3.0	14.5	29.0	1.5	0.9	11.5	51.7	64.1	9.9	6.2
II	T, B=20, ρ=0.9	Linear	3.8	20.9	45.7	2.3	1.9	10.2	64.9	70.6	7.7	5.0
II	T, B=20, ρ=0.9	Spline	15.8	32.1	54.6	2.3	1.9	14.2	64.7	70.5	7.7	5.0
III	T, B=20, ρ=0.9	Linear	5.2	23.9	49.7	3.2	1.4	7.3	60.7	63.5	5.6	3.1
III	T, B=20, ρ=0.9	Spline	19.0	35.1	60.6	3.2	1.4	10.6	61.7	65.5	5.6	3.1
IV	T, B=20, ρ=0.9	Linear	3.6	6.6	7.5	2.4	1.0	56.1	72.2	67.5	54.6	44.3
IV	T, B=20, ρ=0.9	Spline	8.6	7.5	8.0	2.4	1.0	58.4	72.0	67.2	54.6	44.3
V	T, B=20, ρ=0.9	Linear	3.7	7.9	22.0	3.5	1.1	68.7	82.7	65.5	59.6	46.3
I	T, B=10, ρ=0.2	Linear	1.8	9.9	7.8	1.6	0.8	8.3	51.3	57.2	8.0	5.9
II	T, B=10, ρ=0.2	Linear	3.4	15.0	8.1	3.4	1.5	7.3	63.1	61.3	6.4	4.3
II	T, B=10, ρ=0.2	Spline	4.0	16.7	9.9	3.4	1.5	8.6	63.2	61.5	6.4	4.3
III	T, B=10, ρ=0.2	Linear	2.2	15.2	9.5	1.6	1.2	3.7	58.7	59.4	3.0	1.9
III	T, B=10, ρ=0.2	Spline	2.7	18.0	12.7	1.6	1.2	4.2	58.5	59.7	3.0	1.9
IV	T, B=10, ρ=0.2	Linear	2.6	5.5	2.8	2.4	1.0	54.8	71.5	64.6	53.9	43.9
IV	T, B=10, ρ=0.2	Spline	3.0	5.6	2.8	2.4	1.0	55.4	71.5	64.7	53.9	43.9
V	T, B=10, ρ=0.2	Linear	2.7	5.9	22.7	3.0	1.2	69.7	82.8	68.8	60.3	48.0
I	T, B=10, ρ=0.5	Linear	2.2	13.5	14.2	1.6	0.9	9.3	50.8	57.4	8.5	6.1
II	T, B=10, ρ=0.5	Linear	3.3	19.2	13.6	3.4	1.7	7.9	63.1	61.2	7.0	4.4
II	T, B=10, ρ=0.5	Spline	6.2	27.6	21.3	3.4	1.7	9.9	63.5	61.3	7.0	4.4
III	T, B=10, ρ=0.5	Linear	2.3	23.4	21.5	1.3	0.7	4.4	58.0	59.5	3.0	2.1
III	T, B=10, ρ=0.5	Spline	3.8	35.9	31.4	1.3	0.7	5.6	58.1	60.1	3.0	2.1
IV	T, B=10, ρ=0.5	Linear	3.1	6.1	3.4	2.5	1.0	54.4	71.4	63.5	53.4	43.2
IV	T, B=10, ρ=0.5	Spline	4.3	6.6	3.8	2.5	1.0	55.3	71.2	64.0	53.4	43.2
V	T, B=10, ρ=0.5	Linear	3.2	6.9	24.6	3.2	1.3	69.5	82.4	69.0	60.0	47.5
I	T, B=10, ρ=0.9	Linear	7.7	23.0	38.0	1.6	1.0	14.9	51.5	70.9	11.4	6.7
II	T, B=10, ρ=0.9	Linear	10.1	31.5	50.0	4.1	1.7	12.4	65.4	76.2	11.1	6.0
II	T, B=10, ρ=0.9	Spline	41.7	43.6	60.7	4.1	1.7	22.4	68.2	78.9	11.1	6.0
III	T, B=10, ρ=0.9	Linear	12.7	36.2	62.9	2.2	1.3	11.0	60.5	77.2	5.8	2.6
III	T, B=10, ρ=0.9	Spline	43.0	48.4	71.0	2.2	1.3	19.3	62.9	78.7	5.8	2.6
IV	T, B=10, ρ=0.9	Linear	6.2	9.2	11.1	3.2	1.0	56.3	72.1	68.3	54.2	42.4
IV	T, B=10, ρ=0.9	Spline	15.1	10.8	11.8	3.2	1.0	59.3	71.8	68.3	54.2	42.4
V	T, B=10, ρ=0.9	Linear	6.6	10.3	22.5	4.6	1.2	69.6	83.0	67.2	60.3	45.9

S7 Supplementary figures

Figure S1: Simulation results for m=1,000 features and t-distributed independent test statistics showing the true function $\pi_0(\mathbf{x}_i)$ in black and the empirical means of $\hat{\pi}_0(\mathbf{x}_i)$, assuming different modelling approaches in orange (for our approach, Boca-Leek = BL), blue (for the Scott approach with the theoretical null = Scott T), and brown (for the Storey approach.) The scenarios considered are those in Figure 3.

(a)

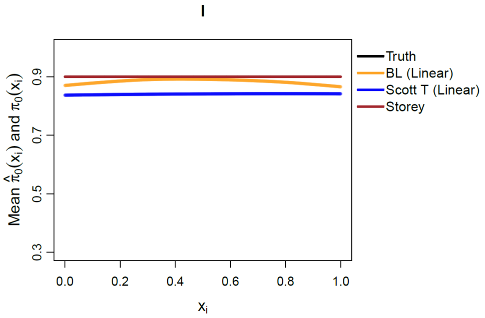

(b)

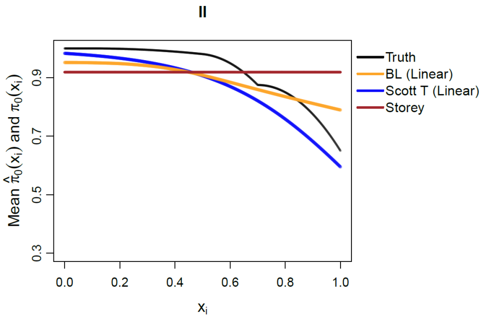

(c)

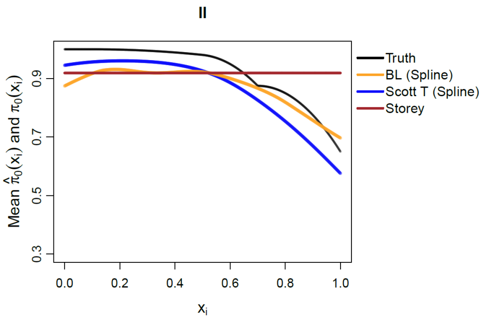

(d)

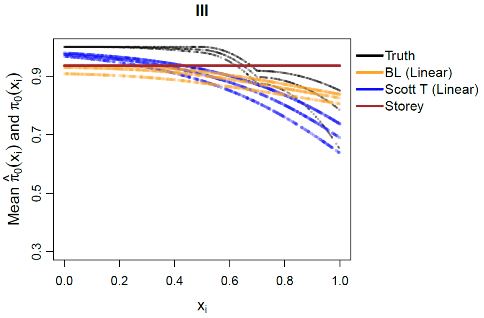

(e)

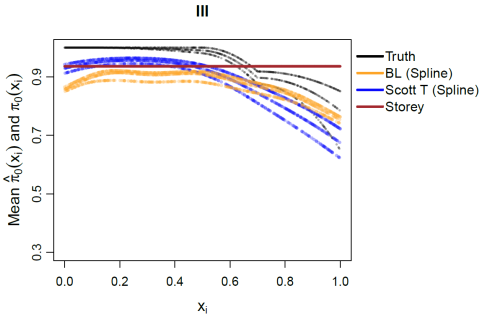

(f)

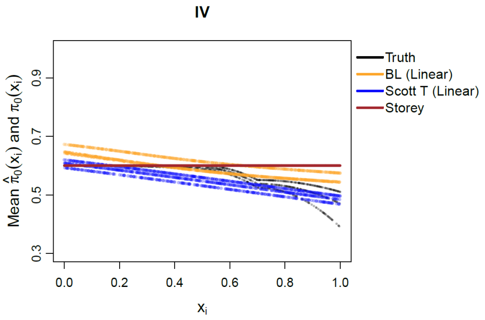

(g)

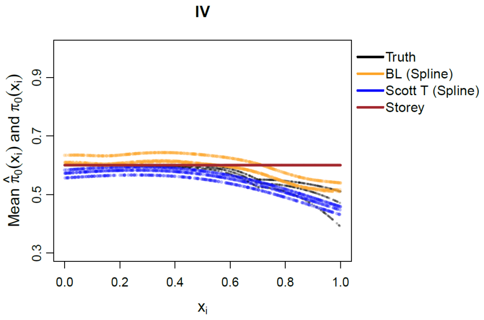

(h)

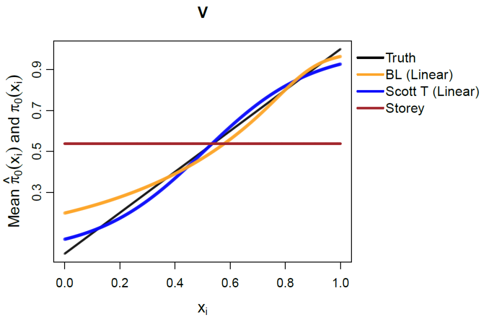

Figure S2: Simulation results for m=10,000 features and t-distributed independent test statistics showing the true function $\pi_0(\mathbf{x}_i)$ in black and the empirical means of $\hat{\pi}_0(\mathbf{x}_i)$, assuming different modelling approaches in orange (for our approach, Boca-Leek = BL), blue (for the Scott approach with the theoretical null = Scott T), and brown (for the Storey approach.) The scenarios considered are those in Figure 3.

(a)

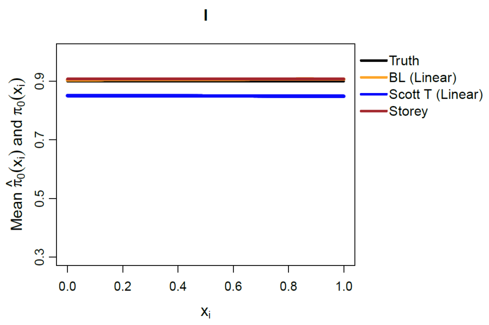

(b)

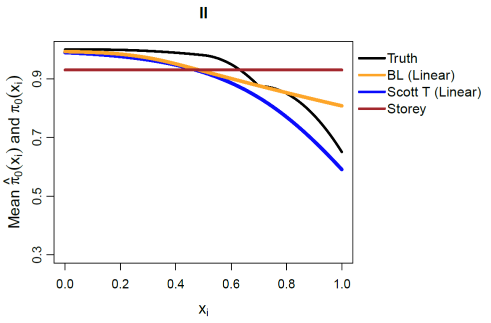

(c)

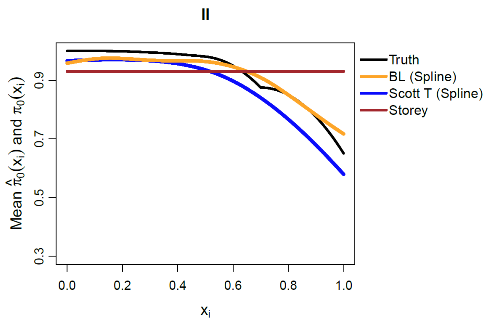

(d)

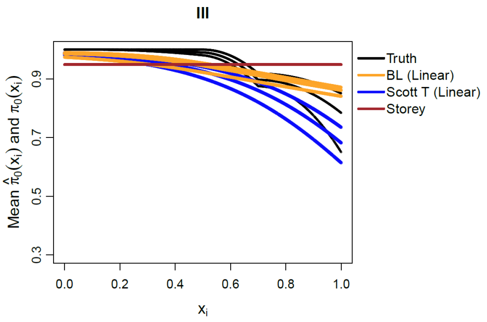

(e)

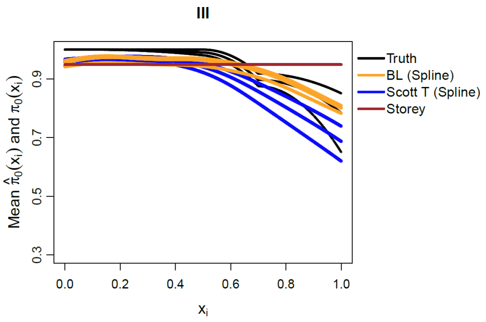

(f)

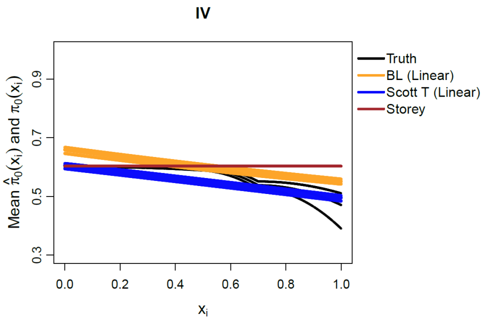

(g)

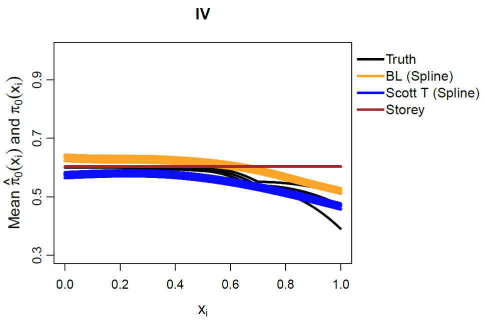

(h)

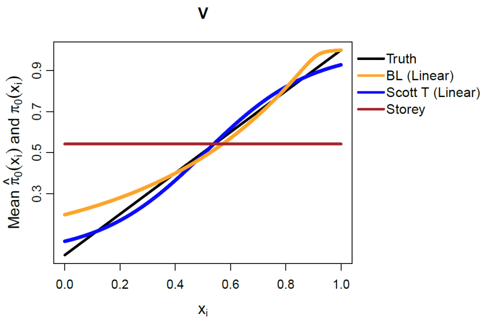

Figure S3: Diagnostic plots for assessing whether, in the BMI GWAS meta-analysis, the p-values and the covariates are conditionally independent under the null. Panel a) stratifies according to N, splitting up the dataset into 8 approximately equal datasets, panel b) uses the MAF stratification.

(a) Stratification by N

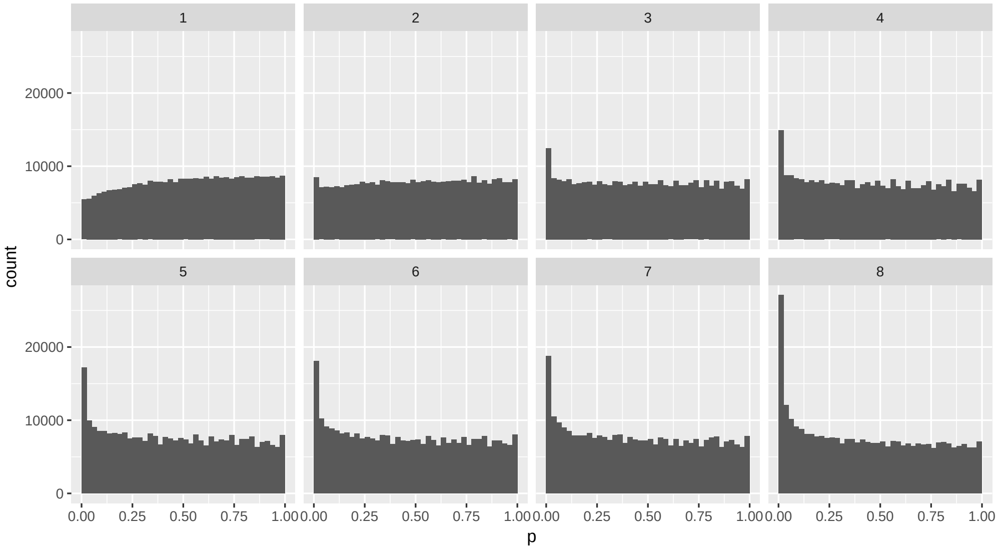

(b) Stratification by MAF

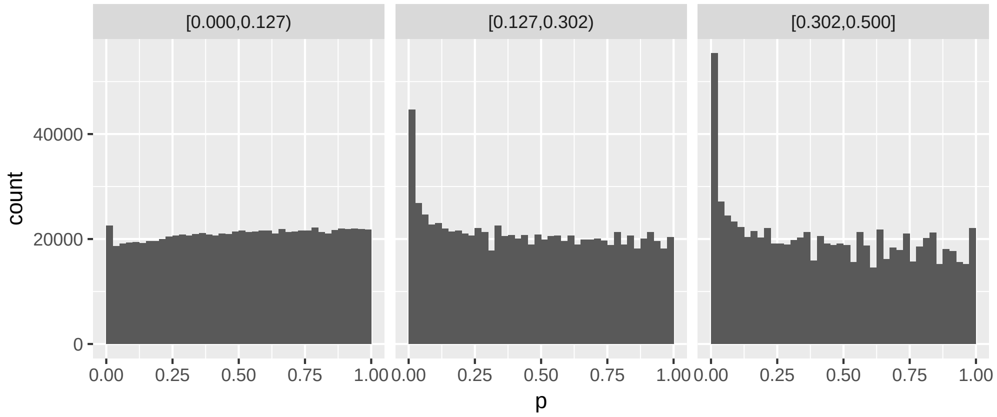

Supplement: Supplemental Information 1 [file peerj-06-6035-s001.pdf]
